# The Effects of a Multi-Ingredient Performance Supplement on Hormonal Profiles and Body Composition in Male College Athletes

**DOI:** 10.3390/sports4020026

**Published:** 2016-05-06

**Authors:** Matthew H. Sharp, Kevin A. Shields, Jacob T. Rauch, Ryan P. Lowery, Shane E. Durkee, Gabriel J. Wilson, Eduardo O. De Souza

**Affiliations:** 1 Applied Science and Performance Institute, Tampa, FL 33607, USA; ryanplowery@gmail.com; 2 Department of Health Sciences and Human Performance, The University of Tampa, Tampa, FL 33606, USA; kevinshields31@gmail.com (K.A.S.); jacobrauch1@gmail.com (J.T.R.); edesouza@ut.edu (E.O.D.S.); 3 Maximum Human Performance, West Caldwell, NJ 07006, USA; sdurkee@maxperformance.com (S.E.D.); gwilson@maxperformance.com (G.J.W.)

**Keywords:** lean body mass, resistance training, testosterone, herbal ingredients

## Abstract

Periods of intense training can elicit an acute decline in performance and body composition associated with weakened hormone profiles. This study investigated the effects of a multi-ingredient performance supplement (MIPS) on body composition and hormone levels in college athletes following a six-week training protocol. Twenty male college athletes were equally assigned to MIPS and placebo (PLA) groups for supplementation (three pills, twice daily) in conjunction with resistance training and specialized sports training (e.g., nine total sessions/week) for six weeks. Dual Energy X-ray Absorptiometry determined body composition at weeks 0 and 6. Serum samples collected at weeks 0 and 6 determined free testosterone (FT), total testosterone (TT), IGF-1 and total estrogen (TE) levels. PLA experienced a significant decline in lean body mass (LBM) (−1.5 kg; *p* < 0.05) whereas the MIPS sustained LBM. The MIPS increased TT 21.9% (541.5 ± 48.7 to 639.1 ± 31.7) and increased FT 15.2% (13.28 ± 1.1 to 15.45 ± 1.3 ng/dL) (*p* < 0.05). Conversely, PLA decreased TT 7.9% (554.5 ± 43.3 to 497.2 ± 39.1 ng/dL), decreased FT 17.4% (13.41 ± 1.8 to 11.23 ± 2.55 ng/dL), and decreased FT:E 12.06% (*p* < 0.05). These findings suggest the MIPS can prevent decrements in LBM and anabolic hormone profiles during intense training periods.

## 1. Introduction

Testosterone is an anabolic, androgenic steroid hormone, which, like other steroid hormones, is derived from cholesterol. In addition, higher testosterone levels have been demonstrated to contribute to increased muscle growth and activation of the nervous system, resulting in enhanced power and strength, mood, libido, and several other benefits [1]. On the other hand, the current literature suggests that as little as a 10% decline in testosterone concentration can significantly reduce gains in strength and muscle hypertrophy [2]. Interestingly, there are conflicting results regarding the anabolic role of testosterone in strength training-induced adaptations [3,4] while other studies have demonstrated positive association of testosterone response on functional performance and body composition [5,6].

Despite the aforementioned conflicts, a growing interest in herbal ingredients to increase testosterone levels among athletic populations remains. However, with treatment or supplementation with multi-ingredient performance supplements (MIPS) that contain multiple herbal ingredients (*i.e.*, fenugreek and tribulus terrestris) said to improve the testosterone profile, there is concern with the excess production of endogenous testosterone metabolites such as estrogen and dihydrotestosterone (DHT). These metabolites can cause side effects and also operate in a negative feedback loop by inhibiting the body’s natural production of luteinizing hormone and testosterone [7].

To date, no research exists to examine the interaction of MIPS on hormone levels, body composition and blood and urine clinical markers in college athletes. Therefore, the first purpose of this study was to examine the efficacy of a MIPS combined with regular training on lean body mass and hormonal profiles in collegiate athletes. In addition, research shows that imbalances in training load and recovery may impair hormonal levels. For instance, there is a significant negative linear correlation (*i.e.*, *r* = −0.60, *p* < 0.01) between overtraining and salivary testosterone concentration [8]. Studies have also found a linear, negative relationship between increased training volume in endurance athletes and testosterone levels [9]. These findings suggest that athletes are at risk of suffering declines in anabolic hormone levels and performance during a competitive season in which high levels of fatigue brought on by high physical demands are likely prevalent. Therefore, the second purpose of this study was to investigate the effects of a MIPS on hormone levels during high levels of specialized sports training combined with resistance training.

## 2. Materials and Methods

### 2.1. Experimental Design

This study utilized a randomized, double-blind, placebo-controlled parallel design to test the effects of a multi-ingredient sports supplement (MHP’s T-BOMB 3XTREME Maximum Human Performance, LLC, 165 Clinton Road, West Caldwell, NJ 07006) (Supplementary Table S1) on hormone profiles and body composition in college athletes during season. Baseline testing prior to supplementation included assessments of body composition via Dual-Energy X-ray Absorptiometry (DEXA), hormone, serum and blood clinical profiles by venipuncture techniques and urine samples for analysis. Following baseline testing, subjects were stratified according to body composition and age and then randomly assigned the placebo (PLA) or MIPS such that baseline values for these parameters were not statistically different. Subjects performed a high frequency training cycle which consisted of four resistance training sessions and five specialized sports training sessions each week for six weeks. All workout sessions were supervised by trained personnel. During the study, both groups consumed three pills with the breakfast meal and three pills post-workout, or with dinner on non-training days. Subjects were retested post-supplementation (week 6) in a manner identical to baseline testing.

### 2.2. Subjects

Twenty college male athletes aged 21 ± 2 years who had a minimum of three years of experience in resistance training volunteered for this study. Subjects could not participate if they were currently taking any medications including anti-inflammatory agents, any performance-enhancing supplements, if they smoked, or if they had any respiratory or metabolic disorders. In an effort to control post-workout nutrition, all participants were supplementing with one serving (1 g CHO, 0 g Fat, 25 g PRO) of whey protein isolate after resistance training. This was considered in their macronutrient profile. Each participant signed an informed consent approved by the Institutional Review Board before participating in the study. The baseline characteristics for each group are provided in Table 1.

### 2.3. Dietary Records

Subjects were required to perform a three-day dietary recall including two weekdays and one weekend day, while none of the three days used were in succession of one another during weeks 0 and 6. The dietary recalls were assessed using a free mobile app (MyFitnessPal, Inc, San Francisco, CA, USA) to determine the average daily macronutrient intake of carbohydrate (CHO), fat, and protein (PRO) to the nearest gram. Each subject recorded their dietary logs at the necessary times for 100% compliance.

### 2.4. Training

The resistance training program was designed to train all major muscle groups centered on compound movements for the upper and lower body. Subjects trained on Monday, Tuesday, Thursday, and Friday of each week. Table 2 contains the specifics of exercise selection, sets, repetitions and rest periods. Monday and Tuesday consisted of hypertrophy training while Thursday and Friday was strength. Training loads were progressively increased by 2%–5% for a given exercise if the subject could lift the target repetitions with proper technique prior to muscular failure. In the event in which target repetitions could not be lifted, the load was decreased by 5%–10% until the repetition scheme was reached. Additionally, all subjects participated in specialized sports training sessions (3–4 h) on days Monday through Friday. In conjunction with resistance training and specialized sports training, all subjects trained, on average, nine times per week. All training sessions were supervised to ensure proper technique, maximal effort and intensity.

### 2.5. Body Composition

A whole body DEXA (Hologic, Bedford, MA, USA) scan was utilized to measure body composition. Lean body mass (LBM) and fat mass (FM) were determined for the total body with the subject laying in a supine position with the knee extended and instructed not to move for the entire duration of the scan (~10 min). Results from each scan were uploaded and accessed on a computer directly connected to the DEXA device. All DEXA scans were conducted from baseline and after completion of the study following a 10 h overnight fast. The coefficient of variation (CV) for body composition assessment was 1.5%.

### 2.6. Blood Collection

All blood samples were collected at baseline and at the end of week 6 via venipuncture by a trained phlebotomist. All samples were collected after a 10 h overnight fast at the same time range (0700–1000 h). After coagulation, blood was centrifuged at 1500 *g* for 15 min at 4 °C. Serum was aliquoted and stored at −80 °C for further analysis.

### 2.7. Statistical Analysis

Normality was confirmed by visual inspection of box plots and Shapiro Wilk test. A two-way ANOVA for repeated measures was performed for each dependent variable, assuming supplementation (PLA and MIPS) and time (pre and post) as fixed factors (GraphPad Prism 6, La Jolla, CA, USA). Whenever a significant F-value was obtained, a *post-hoc* test with Bonferroni’s adjustment was used for multiple comparison purposes. The significance level was set at *p* < 0.05. Results are expressed as mean ± standard error.

## 3. Results

### 3.1. Dietary Analysis

There were no significant differences between groups in total caloric or macronutrient intake of carbohydrate (CHO), fat, or protein (PRO) over the course of the three-day dietary recall at week 0 or week 6 (Table 3; *p* > 0.05).

### 3.2. Body Composition

**Figure 1:**
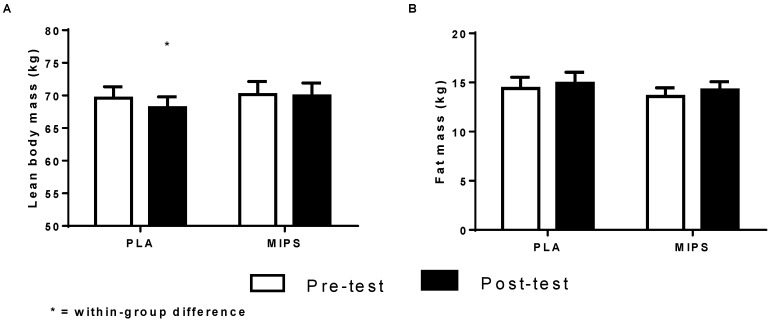
Body composition. (**A**) Group pre- and post-test lean body mass values; (**B**) Group pre- and post-testing fat mass values.

There were no significant differences for TM and FM after six weeks for PLA and MIPS groups (*p* > 0.05). There was a significant group × time interaction for LBM in which the PLA group experienced a decline in LBM (−1.5 kg) (*p* < 0.05) after six weeks (Figure 1).

### 3.3. Hormone Profiles

**Figure 2:**
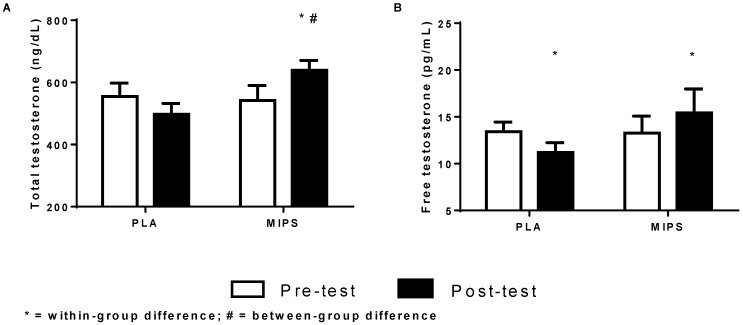
Measures of Plasma Total & Free Testosterone. (**A**) Group pre- and post-test total testosterone levels; (**B**) Group pre- and post-testing free testosterone levels.

**Figure 3:**
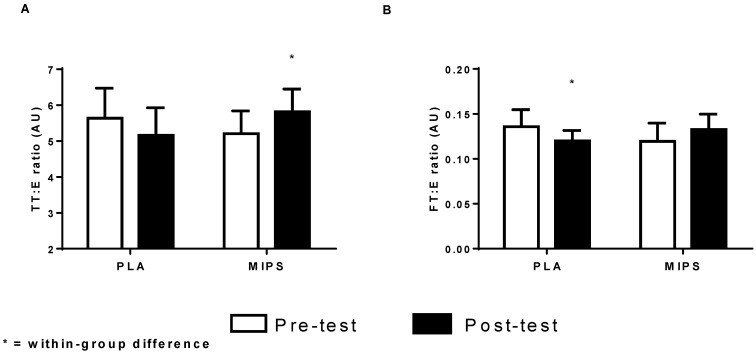
Total Testosterone to Estrogen (TT:E) & Free Testosterone to Estrogen (FT:E) Ratios, (**A**) Group pre- and post-testing TT:E values; (**B**) Group pre- and post-testing FT:E values.

There was a significant group × time interaction (*p* < 0.0007) favoring an increase in total testosterone for the MIPS group. In addition, the pairwise comparison revealed between-group differences at week 6 (MIPS: 21.9% *vs.* PLA: −7.9%; *p* < 0.03). Likewise, there was a significant group × time interaction (*p* < 0.0006) in which MIPS increased free testosterone 15.2% (*p* < 0.01) and the PLA group decreased −17.4% (*p* < 0.01) (Figure 2). The pairwise comparison did not reveal a between-group difference (*p* > 0.05). Results for total and free testosterone to estrogen ratios (TT:E and FT:E) are presented in Figure 3A and 3B, respectively. No significant between-groups differences were detected prior to the experimental period (*p* > 0.05). There was a significant group × time interaction for TT:E (*p* < 0.006) (Figure 3). Pairwise comparisons revealed that only the MIPS group significantly increased TT:E 11.71% (*p* < 0.05), whereas PLA tended to have declined in TT:E by −8.41% (*p* = 0.072). Likewise, there was a significant group × time interaction for FT:E (*p* < 0.003) in which the pairwise comparisons revealed that the MIPS group increased 10.51% (*p* < 0.05), whereas the PLA significantly decreased −12.06% (*p* < 0.03).

### 3.4. Clinical Safety Data

There were no group × time interactions or main effects (*p* > 0.05) observed in any whole blood, serum or urinary clinical safety markers tested (Supplementary Tables S2–S4).

## 4. Discussion

The current study’s purpose was two-fold: (I) to examine the efficacy of a MIPS on lean body mass and hormonal profiles in college athletes during the season; and (II) to investigate the effects of a MIPS on hormone levels during high levels of specialized sports training combined with resistance training. The main finding of the current study was that MIPS staved off the declines seen in LBM and testosterone during high intensity training. These findings have broad implications for athletes seeking to optimize testosterone levels and maintain LBM during training seasons.

Athletes often undergo periods of rigorous training in order to compete or prepare for competitions. It is important to mention that athletes in this trial trained, on average, nine times per week throughout the duration of the study, combining both resistance training and specialized sports training. Furthermore, only the PLA group demonstrated significant declines in total and free testosterone as well as LBM. However, the MIPS group sustained LBM associated with positive changes in hormonal profile. This beneficial effect observed in the MIPS group could be associated with the fact that the MIPS used in this study contains multiple ingredients which may have contributed to elevated total and free testosterone as observed in Figure 2A,B. For instance, key ingredients contained in the MIPS have been shown to improve free and total testosterone. Among them are herbal plant extracts fenugreek [10], tribulus and tongkat ali [11], as well as the minerals zinc and magnesium aspartate (ZMA) [12] (Supplementary Table S1). While the dose of these ingredients is proprietary, they approximate the clinical doses used in previous research which have shown that a range of 300–600 mg of fenugreek [10] as well as tribulus [13], 50 mg of zinc [14,15], and 30 mg of magnesium can support testosterone levels [16]. Ingredients in the MIPS such as stinging nettle leaf are known for increasing the amount of free (active) testosterone by binding the testosterone inhibitor sex hormone binding globulin (SHBG) [17]. In addition, the effects of ingredients in the investigated MIPS on LBM mass and hormone profiles are controversial. Some studies have demonstrated positive adaptations while other trials have not demonstrated any treatment effect on hormone profiles and LBM [11,12,17,18,19,20,21,22,23,24,25,26]. Moreover, some studies have demonstrated that testosterone might induce muscle growth in specific situations [27], but the post-exercise hormone responses are still not associated with a higher protein synthesis rate or muscle hypertrophy [28]. However, it is important to mention that the present study was the first to investigate the effects of MIPS in college athletes during an intense training phase (*i.e.*, nine sessions/week). Thus, our results suggest that athletic populations might have positive outcomes when supplementing with a MIPS that claims to improve hormone profiles. Furthermore, additional research is necessary to understand the mechanism for producing such outcomes.

Another important finding of the current study was that no significant elevation of plasma estrogen levels occurred in the MIPS group. It has been demonstrated that elevations in testosterone caused by drug therapy have caused deleterious side effects. Included among these side effects are increased estrogen and DHT as well as a decline in natural testosterone production [7]. In this study, the MIPS significantly increased testosterone with no change in estrogen levels. The ability of the MIPS to attenuate a rise in estrogen may be due to multiple ingredients including chrysin [29], DIM (diindolymethane) [26] and stinging nettle leaf [17]. These extracts have been demonstrated to attenuate the activity of aromatase and also compete for estrogen at its receptor binding site.

Finally, there were no adverse side effects reported from the participants and no significant changes in hemodynamic measures and in clinical chemistry markers measured in whole blood, serum, or urine, suggesting that the MIPS at the investigated dosages for a period of six weeks appears safe (Supplementary Tables S2–S4).

## 5. Conclusions

The collective results of this research indicate that a MIPS used in the current study results in positive hormonal profiles while sustaining lean body mass in college athletes after six weeks of training with a high frequency (nine sessions/week) protocol compared to a visually identical, volume-matched placebo. Therefore, our findings suggest that athletes and resistance-trained individuals may avoid lean body mass loss during high intensity training cycles by supplementing with MIPS formulas. In addition, further studies are needed to elucidate the athletic performance–related effects and the mechanisms of action of MIPS in sustaining lean mass in trained populations, as this manuscript lacks any performance data. Moreover, these results should be further examined to discover if MIPS can be used to help other populations which seek to sustain or improve lean body mass during catabolic conditions combined with resistance training. The effects of the MIPS in female athletes also warrants further examination. It is important to note that catabolic conditions (e.g., LBM decrements) should be avoided by monitoring an athlete’s recovery and decreasing physical stress in order to optimize on-field performance.

## Figures and Tables

**Table 1:** Physical baseline characteristics of participants.

Characteristic	PLA (*n* = 10)	MIPS (*n* = 10)
Age (years)	21 ± 0	21 ± 2
LBM (kg)	69.58 ± 1.75	70.13 ± 2.01
FM (kg)	14.40 ± 1.12	13.58 ± 1.00
TM (kg)	83.98 ± 2.16	83.71 ± 1.87
BF%	17.0 ± 1.1	16.2 ± 1.1

The baseline characteristics expressed as mean ± standard error for both groups are shown in Table 1. There were no between-group difference for any variable at week 0 of the study (*p* > 0.05). LBM = Lean Body Mass; FM = Fat Mass; TM = Total Mass; BF % = Body Fat Percent; kg = kilograms.

**Table 2:** Resistance training protocol.

Day 1 and 3				Day 2 and 4			
Exercise	Sets	Reps	Rest	Exercise	Sets	Reps	Rest
Banded BB Back Squat	5	3	3 min	Banded BB Deadlift	4	2	3 min
DB Hip Bridge	3	10	1–2 min	(1) Supinated Pull-Up	3	5	1–2 min
(1) BB Bent Row	3	6	1–2 min	(2) DB Romanian Deadlift	3	5	
(2) BB Lateral Lunge	3	6		(3) Cable Row	3	12	
(1) Neutral Grip DB Press	3	6	1–2 min	(1) BB Split Squat	2	6	1–2 min
(2) Cable Paloff Press	3	10		(2) Unilateral DB Row	2	6	
BB Reverse Lunge	2	8	1–2 min	(1) DB Lateral Step-Up	2	6	1–2 min
				(2) DB Cuban Press			

BB = barbell; DB = dumbbell. Numbered exercises indicate that the movements were performed subsequently without rest, then rested after performing the final numbered movement.

**Table 3:** Three-day dietary recalls for PLA and MIPS groups at weeks 0 and 6.

Dietary Variable	PLA		MIPS	
	Week 0	Week 6	Week 0	Week 6
CHO (g/kg)	4.3 ± 0.5	4.3 ± 0.4	4.3 ± 0.4	4.4 ± 0.4
Fat (g/kg)	1.0 ± 0.1	1.0 ± 0.1	0.9 ± 0.1	1.0 ± 0.1
PRO (g/kg)	1.9 ± 0.1	2.0 ± 0.1	2.0 ± 0.2	2.1 ± 0.2
Total kcals	2832 ± 40	2857 ± 60	2818 ± 41	2925 ± 58

No significant differences were observed between groups for total calories or macronutrient intakes (*p* > 0.05).

## Supplementary Materials

Supplementary materials can be accessed at: http://www.mdpi.com/2075-4663/4/2/26/s1.
