# Supplementary material for: The Effects of a Multi-Ingredient Performance Supplement on Hormonal Profiles and Body Composition in Male College Athletes
Source: Sports (Basel). 2016 May 6;4(2):26. doi: 10.3390/sports4020026 (PMC5968923; doi:10.3390/sports4020026)
Supplement: Supplementary file 1 [file sports-04-00026-s001.pdf]

SupplementaryMaterials

The Effects of a Multi-Ingredient Performance Supplement on Hormonal Profiles and Body Composition in Male College Athletes

Matthew H. Sharp [1,*], Kevin A. Shields [2,†], Jacob T. Rauch [2,†], Ryan P. Lowery [1,†], Shane E. Durkee [3,†], Gabriel J. Wilson [3,†] and Eduardo O. De Souza [2,†]

Table S1. MIPS Proprietary Blend.

**1709 mg Per Serving** [+]
TRIBULUS EXTRAT (WHOLE PLANT) (Tribulu terrestris) (15% protodioscin), FENUGREEK EXTRACT (Trigonella foenum-graecum) (4:1 EXT), FLAXSEED POWDER (Linum usitatissimum), CHRYSIN, KUDZU EXTRACT (ROOT) (Pueraria lobata), RED CLOVER EXTRACT (ARIAL PARTS) (Trifolium pratense), ZINC SULFATE MONOHYDRATE, LONGJACK EXTRACT (ROOT) (Eurycoma longifolia jack), GLYCINE, L-ARGININE HCL, MAGNESIUM OXIDE, DI INDOLYL METHANE, L-METHIONINE, COPPER GLUCONATE, CORDYCEPS EXTRACT (WHOLE PLANT) (Cordyceps sinensis), MIXED PLANT PHYTOSTEROLS, NETTLE EXTRACT (LEAF) (Urtica dioica), OAT STRAW EXTRACT (AERIAL PARTS) (Avena sativa L), SAW PALMETTO EXTRACT (FRUIT), (Serenoa repens), BIOPERIN, NADH

[+] Subjects took two servings of the MIPS proprietary blend daily.

Table S2. Whole Blood Clinical Chemistry Markers.

**Variable**	**Placebo**		**MIPS**		**G x T *p*-Value**
	**Pre**	**Post**	**Pre**	**Post**	
WBC (K/μL)	5.9 ± 1.2	6.1 ± 1.0	5.7 ± 0.7	5.5 ± 0.5	0.106
RBC (M/μL)	4.6 ± 0.9	5.0 ± 0.5	4.9 ± 0.1	4.8 ± 0.1	0.245
Hemoglobin (g/dL)	15.5 ± 1.1	15.7 ± 1.1	14.6 ± 0.5	14.7 ± 0.4	0.639
Hematocrit (%)	45.2 ± 3.6	45.2 ± 3.1	43.4 ± 1.0	43.7 ± 1.7	0.878
MVC (fL)	91 ± 5	89 ± 5	89 ± 3	90 ± 4	0.061
MCH (pg)	31.0 ± 1.4	30.4 ± 1.7	30.8 ± 1.2	30.5 ± 1.2	0.557
MCHC (g/dL)	33.9 ± 0.7	34.3 ± 0.9	33.3 ± 0.9	33.4 ± 0.9	0.523
Neutrophils (K/μL)	2.9 ± 1.2	3.5 ± 1.6	3.2 ± 0.5	3.0 ± 0.4	0.078
Lymphocytes (K/μL)	2.3 ± 0.2	2.1 ± 0.4	2.0 ± 0.3	1.9 ± 0.4	0.617
Monocytes (K/μL)	0.6 ± 0.1	0.5 ± 0.1	0.5 ± 0.1	0.5 ± 0.1	0.850
Eosmophils (K/μL)	0.2 ± 0.1	0.2 ± 0.1	0.2 ± 0.1	0.2 ± 0.1	0.777
Basophils (K/μL)	0.1 ± 0.1	0.1 ± 0.1	0.1 ± 0.1	0.1 ± 0.1	0.584

Values are reported as mean ± standard deviation. WBC = white blood cells; RBC = red blood cells; MVC = mean cell volume; MCH = mean cell hemoglobin; MCHC = mean cell hemoglobin concentration.

Table S3. Serum Clinical Chemistry Markers.

**Variable**	**Placebo**		**MIPS**		**G x T *p*-Value**
	**Pre**	**Post**	**Pre**	**Post**	
Glucose (mg/dL)	89 ± 5	89 ± 5	84 ± 4	84 ± 5	0.296
BUN (mg/dL)	19 ± 4	20 ± 5	19 ± 3	20 ± 4	0.140
Creatinine (mg/dL)	1.1 ± 0.1	1.1 ± 0.1	1.04 ± 0.1	1.04 ± 0.1	0.528
Total Protein (g/dL)	6.8 ± 0.2	6.8 ± 0.2	7.2 ± 0.7	7.1 ± 0.6	0.398
Albumin (g/dL)	4.6 ± 0.2	4.6 ± 0.3	4.6 ± 0.38	4.6 ± 0.2	0.893
Total Bilirubin (mg/dL)	0.4 ± 0.1	0.3 ± 0.1	0.6 ± 0.3	0.5 ± 0.3	0.999
Sodium (mmol/L)	142 ± 2	142 ± 2	140 ± 2	140 ± 2	0.999
Potassium (mmol/L)	4.4 ± 0.2	4.5 ± 0.4	4.1 ± 0.1	4.1 ± 0.2	0.521

Chloride (mmol/L)	102 ± 1	102 ± 1	100 ± 1	101 ± 1	0.838
CO_2 (mmol/L)	24 ± 1	24 ± 2	25 ± 1	25 ± 1	0.697
ALP (IU/L)	88 ± 25	84 ± 24	85 ± 14	83 ± 18	0.094
AST (IU/L)	29 ± 5	30 ± 6	26 ± 3	26 ± 3	0.357
ALT (IU/L)	22 ± 3	22 ± 3	25 ± 4	26 ± 4	0.432

Values are reported as mean ± standard deviation. HDL = high density lipoprotein; LDL = low density lipoprotein; BUN = blood urea nitrogen; CO_2 = carbon dioxide; ALP = Alkaline phosphatase; AST = aspartate aminotransferase; ALT = alanine transaminase.

Table S4. Urine Clinical Markers.

**Variable**	**Placebo**		**MIPS**		**G x T *p*-Value**
	**Pre**	**Post**	**Pre**	**Post**	
Glucose	0.0 ± 0.0	0.0 ± 0.0	0.0 ± 0.0	0.0 ± 0.0	0.999
Ketones	0.0 ± 0.0	0.0 ± 0.0	0.0 ± 0.0	0.0 ± 0.0	0.999
Occult Blood	0.0 ± 0.0	0.0 ± 0.0	0.0 ± 0.0	0.0 ± 0.0	0.999
Protein	0.0 ± 0.0	0.0 ± 0.0	0.0 ± 0.0	0.0 ± 0.0	0.999
Nitrite	0.0 ± 0.0	0.0 ± 0.0	0.0 ± 0.0	0.0 ± 0.0	0.999
Bilirubin	0.0 ± 0.0	0.0 ± 0.0	0.0 ± 0.0	0.0 ± 0.0	0.999
Urobilinogen (mg/dL)	0.3 ± 0.2	0.3 ± 0.2	0.3 ± 0.3	0.3 ± 0.2	0.196
pH	6.0 ± 0.4	6.2 ± 0.3	6.2 ± 0.2	6.1 ± 0.2	0.098
Specific Gravity	1.025 ± 0.008	1.027 ± 0.004	1.019 ± 0.007	1.019 ± 0.009	0.484

Values are reported as mean ± standard deviation. Zeros indicate a lack of detectable variable in urine.
